# Wedelolactone, a medicinal plant-derived coumestan, induces caspase-dependent apoptosis in prostate cancer cells via downregulation of PKCε without inhibiting Akt

**DOI:** 10.3892/ijo.2012.1664

**Published:** 2012-10-16

**Authors:** SIVALOKANATHAN SARVESWARAN, SUBHASH C. GAUTAM, JAGADANANDA GHOSH

**Affiliations:** 1 Vattikuti Urology Institute; 2 Department of Surgery and; 3 Josephine Ford Cancer Center, Henry Ford Health System, Detroit, MI 48202, USA

**Keywords:** wedelolactone, 5-lipoxygenase, protein kinase Cε, prostate cancer, apoptosis

## Abstract

Emerging studies indicate that metabolism of arachidonic acid through the 5-lipoxygenase (5-Lox) pathway plays a critical role in the survival of prostate cancer cells raising the possibility that 5-Lox can be targeted for an effective therapy of prostate cancer. Wedelolactone (WDL), a medicinal plant-derived natural compound, is known to inhibit 5-Lox activity in neutrophils. However, its effect on apoptosis in prostate cancer cells has not been addressed. Thus, we tested the effects of WDL on human prostate cancer cells *in vitro*. We observed that WDL kills both androgen-sensitive as well as androgen-independent prostate cancer cells in a dose-dependent manner by dramatically inducing apoptosis. We also found that WDL-induced apoptosis in prostate cancer cells is dependent on c-Jun N-terminal Kinase (c-JNK) and caspase-3. Interestingly, WDL triggers apoptosis in prostate cancer cells via downregulation of protein kinase Cε (PKCε), but without inhibition of Akt. WDL does not affect the viability of normal prostate epithelial cells (PrEC) at doses that kill prostate cancer cells, and WDL-induced apoptosis is effectively prevented by 5-oxoETE, a metabolite of 5-Lox (but not by 15-oxoETE, a metabolite of 15-Lox), suggesting that the apoptosis-inducing effect of WDL in prostate cancer cells is mediated via inhibition of 5-Lox activity. These findings indicate that WDL selectivity induces caspase-dependent apoptosis in prostate cancer cells via a novel mechanism involving inhibition of PKCε without affecting Akt and suggest that WDL may emerge as a novel therapeutic agent against clinical prostate cancer in human.

## Introduction

Wedelolactone (7-methoxy-5,11,12-trihydroxy-coumestan) is a plant-derived natural product synthesized mainly by members belonging to the Asteraceae family (1,2). A major source of WDL is the plant genus *Eclipta* (or Bhringaraj) which is an acrid, bitter herb medicine traditionally used extensively for hair and skin health and for preventing liver damage due to alcohol overdose and jaundice (1–5). This herb expels intestinal worms, cures cough, prevents inflammation, reduces symptoms of bronchitis and asthma, and is used to alleviate uterine pain after delivery. In addition to its use as folk medicine, it has also been used in the treatment of infective hepatitis in India (2–4), snake venom poisoning in Brazil (6–9) and septic shock in China (10). Active compounds in *Eclipta* were observed to inhibit protease activity as well as the activity of phospholipase A2 (11–14). The coumestan compounds wedelolactone and demethyl-wedelolactone were tested to show anti-hepatotoxic effect in liver cells (2,3). WDL and other compounds from the plant *Wedelia sinensis* have also been reported to block androgen receptor function (15), and to inhibit polymerase activity of hepatitis C virus (16). Interestingly, the coumestan derivative, wedelolactone, has been found to be a potent and selective inhibitor of 5-Lox (IC_50_ ∼2.5 *μ*M) which inhibits 5-Lox activity by an oxygen radical scavenging mechanism (17,18). Thus, WDL has emerged as a candidate drug for prevention as well as treatment of inflammatory diseases and cancer.

Emerging evidence from several studies has revealed that prostate cancer cells continuously generate 5-Lox metabolites and inhibition of 5-Lox by specific inhibitors induces apoptosis both in androgen-sensitive as well as androgen-independent prostate cancer cells (19–25). Apoptosis is prevented by metabolites of 5-Lox, but not by 12-Lox or 15-Lox, suggesting that 5-Lox activity plays an essential role in the viability of prostate cancer cells (20). Inhibition of 5-Lox activates caspases and blocking caspase activity by specific inhibitors prevents induction of apoptosis suggesting that this type of apoptosis is caspase-dependent. It was also observed that inhibition of 5-Lox triggers rapid activation of c-Jun N-terminal kinase (JNK) in prostate cancer cells which is detectable within 1–2 h post-treatment (26). Blocking JNK activity by specific chemical inhibitors prevent 5-Lox inhibition-induced caspase activation as well as apoptotic degradation of nuclear DNA to nucleosomal fragments, suggesting that JNK plays an important role in the apoptosis process. JNK has already been reported to play an important role in apoptosis in various types of cells (27–31). In regard to downstream signaling, recently we found that 5-Lox metabolites signal via an Akt-independent, PKCε-dependent mechanism (32,33). Altogether, these findings demonstrated that 5-Lox activity plays a critical role in the survival of prostate cancer cells and suggested that 5-Lox may be used as a molecular target for prevention and treatment of prostate cancer.

Alongside the use of synthetic inhibitors, screening and testing of compounds from natural sources are becoming more and more popular for obtaining improved solubility, potency, and cancer selectivity. We sought to test natural compound inhibitors of 5-Lox activity for their effects on induction of apoptosis in prostate cancer cells with an intention to find novel agents for prostate cancer therapy. Though the 5-Lox inhibitory effect of WDL is known for a while, its effect on induction of apoptosis in prostate cancer cells and the underlying mechanisms have not been addressed before. Thus, we examined the *in vitro* effects of WDL on a range of human prostate cancer cells. Our results show that WDL strongly affects the viability of both androgen-sensitive (LNCaP) as well as androgen-independent (PC3, DU145) human prostate cancer cells with minimal effect on the viability of normal, non-tumor prostate epithelial cells (PrEC). Moreover, WDL was observed to induce caspase-dependent apoptosis in prostate cancer cells which was associated with dramatic inhibition of PKCε but no inhibition of Akt. Apoptosis was effectively prevented by exogenous metabolites of 5-Lox. These findings indicate that WDL selectivity induces caspase-dependent apoptosis in prostate cancer cells via a novel mechanism involving inhibition of PKCε but without inhibition of Akt and suggest that WDL should be tested further as a novel candidate drug for development of an effective therapy against clinical prostate cancer.

## Materials and methods

### Cell culture and reagents

Human prostate cancer cells (LNCaP, PC3 and DU145) were purchased from American Type Culture Collection (Manassas, VA, USA). Cells were grown in RPMI-1640 medium (Invitrogen, Carlsbad, CA, USA) as described before (20). Normal prostate epithelial cells (PrEC) and the growth medium (PrEGM complete) were purchased from Lonza (Walkersville, MD, USA), polyclonal antibodies against histone H2A.X, phosphohistone H2A.X, c-JNK, phospho-JNK, Akt and phospho-Akt were purchased from Cell Signaling (Danvers, MA, USA). Antibodies against PARP, cyclin D1 and PKCε were purchased from Santa Cruz Biotechnology (Santa Cruz, CA, USA). Anti-β-actin antibody, WDL and ibuprofen were purchased from Sigma Chemical Co. (St. Louis, MO, USA). 5-Oxoeicosatetraenoid (5-oxoETE) and 15-oxoETE were purchased from Cayman Chemicals (Ann Arbor, MI, USA).

### Measurement of cell viability

Prostate cancer cells (4×10^3^ per well) were plated in 96-well plates overnight in RPMI-1640 medium supplemented with 10% FBS. PrEC cells were plated in PrEGM complete medium supplemented with 1% FBS. Then the cells were treated with varying doses of WDL or solvent vehicle (0.2% DMSO) and the plates were incubated for 72 h at 37°C in the CO_2_ incubator. Cell viability was measured using One Solution Cell Titer AQ Assay kit following a protocol supplied by the manufacturer (Promega Corp., Madison, WI, USA).

### Microscopy

LNCaP prostate cancer cells (∼3×10^5^) were plated in RPMI-1640 medium supplemented with 10% FBS overnight onto 60-mm diameter tissue culture plates (Falcon) and allowed to grow for 48 h. On the day of experiment, the spent culture medium was replaced with 2 ml fresh RPMI-1640 medium and the cells were treated with inhibitors. Control cells were treated with solvent only (0.2% DMSO). Photographs were taken with a Nikon digital camera attached to a LEICA fluorescence microscope at magnification, ×400. Image acquisition and data processing were done with a Dell computer attached to the microscope using SPOT-Advanced software.

### Western blot analysis

LNCaP cells (∼3×10^5^) were plated and allowed to grow for 48 h. The old medium was then replaced with 2 ml fresh RPMI-1640 medium and the cells were treated with inhibitors. After treatment, cells were harvested, washed and lysed in lysis buffer (50 mM HEPES buffer, pH 7.4, 150 mM NaCl, 1 mM EDTA, 1 mM orthovanadate, 10 mM sodium pyrophosphate, 10 mM sodium fluoride, 1% NP-40, and a cocktail of protease inhibitors). Proteins were separated by 12% SDS-PAGE and transferred to nitrocellulose membranes. Membranes were blocked with 5% non-fat milk solution and then blotted with appropriate primary antibody followed by peroxidase-labeled secondary antibody. Bands were visualized by enhanced chemiluminescence (Amersham, Rockford, IL, USA).

### Annexin V binding

LNCaP cells (∼3×10^5^) were plated in RPMI-1640 medium and allowed to grow for 48 h. The spent culture medium was replaced with fresh 2 ml RPMI-1640 medium and the cells were treated with WDL or ibuprofen for 24 h at 37°C. Then the cells were treated with FITC-labeled Annexin V and propidium iodide for 15 min in the dark using Annexin V-Binding Detection kit following a protocol supplied by the manufacturer (BD Biosciences, San Jose, CA, USA). After washing, cells were photographed with a Nikon digital camera attached to a LEICA fluorescence microscope at magnification, ×200. Image acquisition and data processing were done with a Dell computer attached to the microscope using SPOT-Advanced software.

### Measurement of caspase activity

LNCaP cells (∼3×10^5^ per plate) were plated in 60-mm diameter plates and treated with inhibitors or solvent vehicle for varying periods of time. Then the cells were lysed in lysis buffer containing 0.2% CHAPS as detergent. Enzymatic activity of caspase-3 in cell lysates was measured colorimetrically by a commercially available kit following methods supplied by the manufacturer (Biomol, Plymouth Meeting, PA, USA).

### DNA fragmentation

Apoptosis was quantitatively measured by detecting degradation of nuclear DNA by sandwich-ELISA. LNCaP cells (∼3×10^5^) were plated in 60-mm diameter tissue culture plates and allowed to grow for 48 h. Cells were then treated either with the experimental agents or solvent vehicle for 24 h. At the end of incubation period, cells were lysed and the degradation of nuclear DNA to nucleosomal fragments was measured by Cell Death Detection ELISA^plus^ as described before (20,26), following instructions supplied by the manufacturer (Roche, Indianapolis, IN, USA).

### Mitochondrial permeability transition (MPT)

LNCaP cells (∼3×10^5^) were plated in RPMI-1640 medium and allowed to grow for 48 h. The spent culture medium was replaced with fresh 2 ml RPMI-1640 medium and the cells were treated with WDL or ibuprofen for 8 h at 37°C. Permeability transition of mitochondria was detected using a kit following manufacturer’s protocol (BD Biosciences) by treating cells with 40 nM Mitotracker red for 30 min at 37°C in the incubator. Hoechst dye 33342 was used to stain the nuclei. After washing, cells were photographed with a Nikon digital camera attached to a Leica fluorescence microscope at magnification, ×400. Image acquisition and data processing were done with a Dell computer attached to the microscope using SPOT-Advanced software.

## Results

### WDL reduces viability of prostate cancer cells in a dose-dependent manner

**Figure 1:**
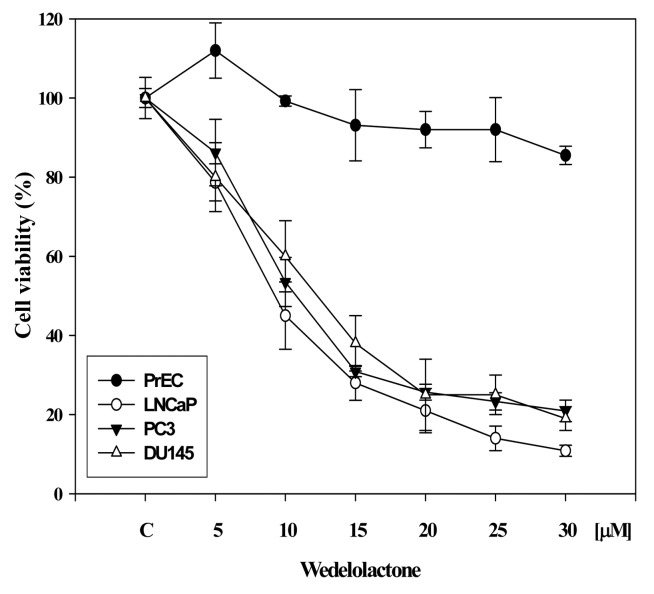
Effect of WDL on the viability of prostate epithelial cells. Cancer and normal prostate epithelial cells (∼4×10^3^ per well) were plated in 96-well plates overnight in complete medium, and treated with varying doses of WDL. Plates were incubated further for 72 h at 37°C and cell viability was measured by Cell Titer assay as described in the Materials and methods section (20,33). Results are shown as mean value of each data point ± SE (n=6). WDL selectively affects the viability of prostate cancer cells sparing normal cells.

Since the role of 5-Lox in the survival and growth of prostate cancer cells has been observed in various laboratories (19–25), we wanted to examine the effect of WDL on the viability of prostate cancer cells, because WDL is known to be a potent inhibitor of 5-Lox activity (17,18). We observed that WDL dose-dependently reduced the viability of both androgen-sensitive (LNCaP) as well as androgen-independent (PC3, DU145) prostate cancer cells with IC_50_s between 8–12 *μ*M (Fig. 1). The effect of WDL was observed to be strongly cancer-specific when compared to its effect on the viability of normal, non-cancerous prostate epithelial cells (PrEC).

### WDL induces severe morphological alteration in prostate cancer cells

**Figure 2:**
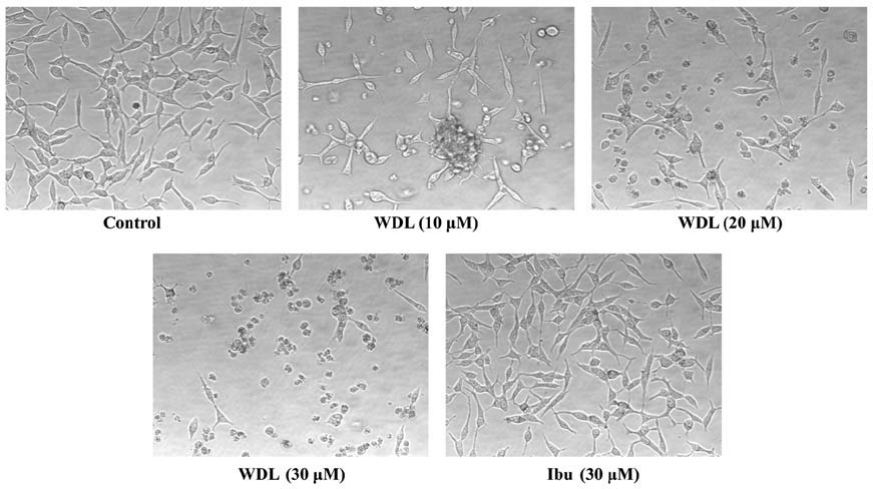
Morphological alteration of LNCaP cells by WDL. LNCaP prostate cancer cells (3×10^5^ per plate) were plated overnight in 60-mm diameter plates as described in the Materials and methods section (Microscopy) and treated either with doses of WDL or ibuprofen for 24 h at 37°C in the incubator. Control cells were treated with the vehicle only (0.2% DMSO). At the end of treatment period, cells were photographed at magnification, ×200. Data show a representative of three experiments with similar results.

Earlier, we reported that prostate cancer cells show pronounced alteration in their morphology forming numerous membrane blebs when treated with synthetic inhibitors of 5-Lox (20). We examined whether WDL also exerts similar effects on prostate cancer cells. We observed that prostate cancer cells treated with WDL show a dramatic alteration in their membrane morphology in a dose-dependent manner. Well-spread adherent cells gradually withdraw their processes, become round and eventually detach and float in the growth medium (Fig. 2). Similar effects of WDL were also observed in PC3 and DU145 cells (not shown). Ibuprofen, an inhibitor of cyclooxygenase, did not show any appreciable effect on the morphology of prostate cancer cells in the same experimental conditions, suggesting a selective action of WDL on these cells. Morphological change of cells with WDL treatment was reminiscent of cells undergoing apoptosis.

### WDL induces apoptosis in prostate cancer cells

**Figure 3:**
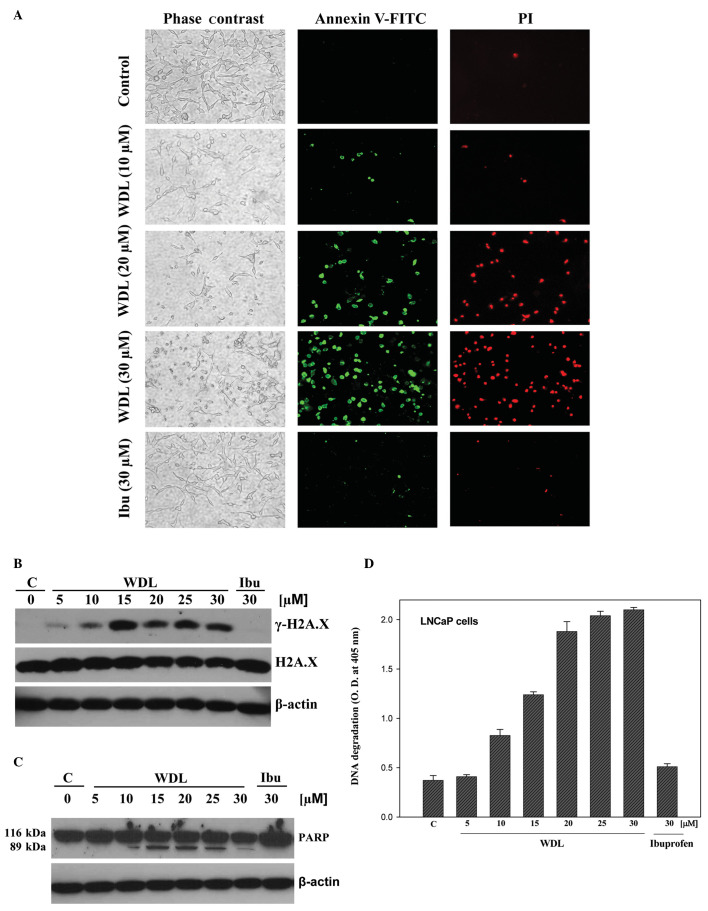
Induction of apoptosis by WDL LNCaP cells (3×10^5^ per plate) were plated as in Fig. 2 above and treated either with WDL or ibuprofen for 24 h. Control cells were treated with 0.2% DMSO. (A) At the end of incubation period, cells were stained with FITC-labeled Annexin V and propidium iodide, and observed under fluorescence microscope at ×200. A representative of two independent experiments is shown here with similar results. (B) At the end of incubation period, cells were lysed and phosphorylation of histone H2A.X at serine-139 was detected by western blot analysis. (C) Cleavage of PARP is shown as detected by western blot analysis. (D) Degradation of chromatin DNA to nucleosomal fragments was detected by Cell Death Detection ELISA. Results are shown as mean values of each data point ± SE (n=4).

WDL-induced morphological alteration in prostate cancer cells prompted us to investigate whether these cells are undergoing death via induction of apoptosis. Apoptosis-associated formation of membrane blebs is characterized by cleavage of cortical cytoskeleton and externalization of phosphatidylserine to the outer leaflet of plasma membrane. Externalization of phosphatidylserine can be assessed by its high-affinity binding with dye-labeled Annexin V. We observed that cells with altered morphology upon WDL treatment bind with fluorescein isothiocyanate-labeled Annexin V (Annexin V-FITC), confirming externalization of phosphatidylserine in these cells after treatment (Fig. 3A).

Ibuprofen did not induce any appreciable effect on externalization of phosphatidylserine in the same experimental conditions. We observed that WDL dose-dependently induced phosphorylation of the DNA damage-indicator histone H2A.X at Serine^139^ (Fig. 3B), suggesting occurrence of DNA strand breaks. Cleavage of poly-ADP ribose polymerase (PARP) is an indicator of advanced stage of apoptosis. PARP is a protein substrate which is cleaved to generate particular peptide fragments by activated caspases. We observed that when prostate cancer cells are treated with WDL, the intact form of PARP protein (molecular weight 116 kDa) is cleaved to generate a characteristic smaller species of ∼89 kDa which was detectable at doses 10 *μ*M and above (Fig. 3C). Degradation of DNA to nucleosomal fragments is an indicator and a well characterized late event in apoptotic cell death. We observed that treatment with WDL induces fragmentation of chromatin DNA to nucleosomes in prostate cancer cells in a dose-dependent manner (Fig. 3D). Ibuprofen did not show any appreciable effect on phosphorylation of H2A.X, cleavage of PARP or degradation of DNA.

### WDL-induced apoptosis in prostate cancer cells is dependent on activation of c-Jun N-terminal kinase (JNK)

**Figure 4:**
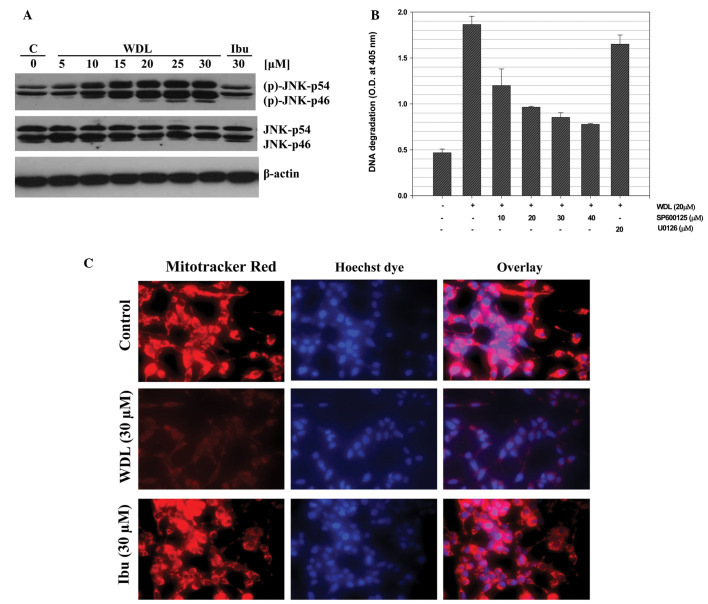
Activation of c-Jun N-terminal Kinase (JNK) and mitochondrial permeability transition by WDL. LNCaP cells (3×10^5^ per plate) were plated as in Fig. 2 and treated with varying doses of WDL or ibuprofen (30 *μ*M) at 37°C for 24 h. (A) Cell lysates were analyzed by western blot analysis with an antibody against phosphorylated-JNK. Antibodies against pan-specific JNK and β-actin were used as controls. A representative of three experiments with similar results is shown here. (B) Role of JNK in apoptosis was tested by pre-treating cells with specific inhibitor, SP600125. U0126, an inhibitor MAPK kinase, was used as negative control. Results are shown as mean values of each data point ± SE (n=4). (C) LNCaP cells were treated with WDL for 8 h and permeability transition of mitochondria was detected by treating cells with 40 nM Mitotracker red for 30 min at 37°C in the incubator. Hoechst dye 33342 was used to stain the nuclei. After washing, cells were photographed with a Nikon digital camera attached to a Leica fluorescence microscope at magnification, ×400.

We previously reported that 5-Lox inhibition induces apoptosis in prostate cancer cells via rapid activation of c-Jun N-terminal kinase (26). We examined whether WDL induces apoptosis in prostate cancer cells via activation of JNK. We observed that when prostate cancer cells are treated with WDL a rapid and strong activation of JNK occurs and that inhibition of JNK blocks induction of apoptosis, suggesting that WDL-induced apoptosis in prostate cancer cells is dependent on JNK activity (Fig. 4A and B). We also observed that WDL damages mitochondrial integrity by inducing permeability transition and loss of membrane potential-sensitive dye (Fig. 4C).

### Induction of apoptosis in prostate cancer cells by WDL treatment is caspase-dependent

**Figure 5:**
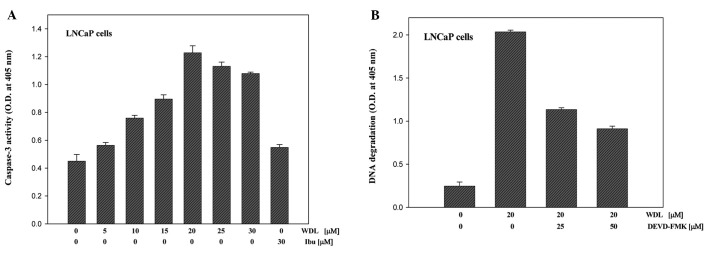
Activation and role of caspase in WDL-induced apoptosis. (A) LNCaP cells (3×10^5^ per plate) were plated as in Fig. 2 above and treated with varying doses of WDL as indicated for 24 h. At the end of incubation period, enzymatic activities of caspase-3 in cell lysates were measured by colorimetric caspase-3 Cellular Activity Assay kit (no. AK-703) using DEVD-pNa as substrate (Biomol). (B) Cells were pretreated for 30 min with specific caspase-3 inhibitor (DEVD-FMK) before treatment with WDL (20 *μ*M) for 24 h. Control cells were treated with the vehicle only (0.2% DMSO). Apoptosis was measured by detecting DNA degradation to nucleosomal fragments by Cell Death Detection ELISA^plus^ (Roche). Data represent mean values of quadruplicate determination of each point ± SE.

Both caspase-dependent and caspase-independent apoptosis are known to occur depending on cell types and apoptotic trigger (27). Though we observed cleavage of PARP (a caspase substrate) after WDL treatment, we wanted to examine the status and role of caspase-3 activation in WDL-induced apoptosis in prostate cancer cells. We observed that treatment with WDL induces activation of caspase-3 in a dose-dependent manner (Fig. 5A). Moreover, we observed that inhibition of caspase-3 by specific inhibitor (DEVD-FMK) significantly prevents apoptotic DNA degradation, suggesting that WDL-induced apoptosis in prostate cancer cells is caspase-dependent (Fig. 5B).

### WDL-induced apoptosis in prostate cancer cells occurs via downregulation of PKCε without inhibiting Akt

**Figure 6:**
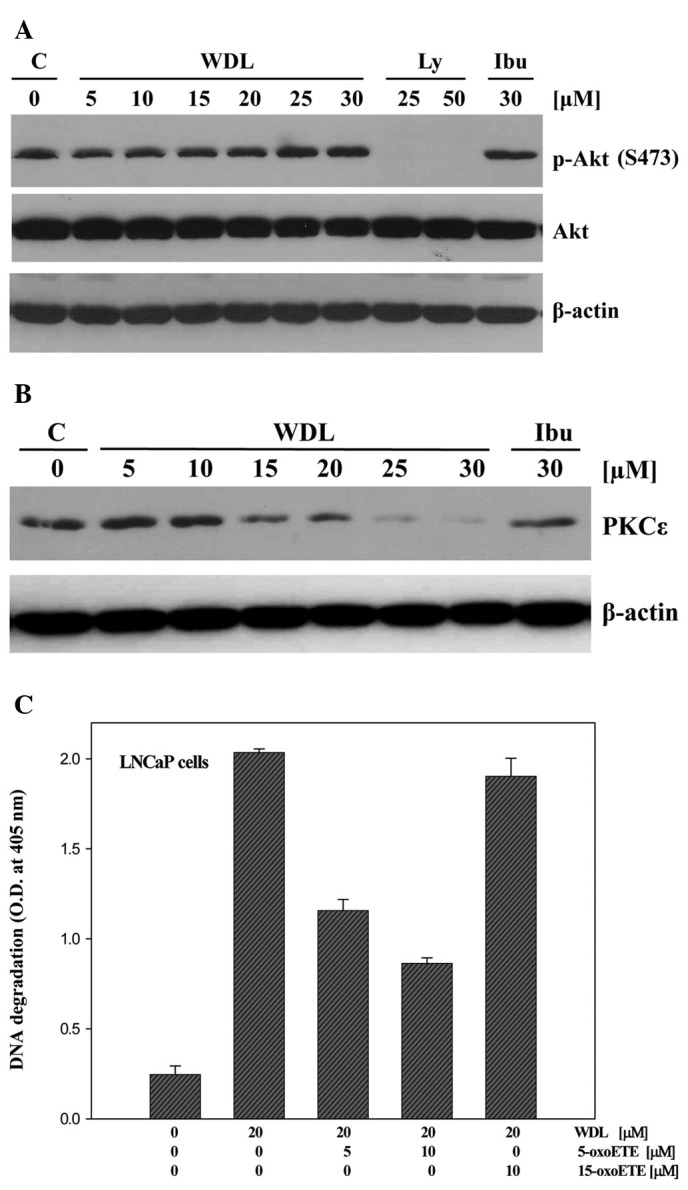
Effect of WDL on Akt and PKCε. (A) LNCaP cells (3×10^5^ per plate) in 60-mm diameter plates were treated with varying doses of WDL for 24 h. Control cells were treated with the solvent vehicle only (0.2% DMSO). Phosphorylation of Akt (at Serine 473) was detected by western blot analysis. LY294002 (LY) and ibuprofen (Ibu) were used as positive and negative controls, respectively. (B) Effect of WDL on the protein level of PKCε was detected by western blot analysis. β-actin was used as loading control. (C) Prevention of WDL-induced apoptosis by 5-oxoETE. LNCaP cells (3×10^5^ per plate) were plated in 60-mm diameter plates and treated with WDL (20 *μ*M) with or without the addition of exogenous 5-oxoETE or 15-oxoETE. Plates were incubated at 37°C for 24 h in the CO_2_ incubator. Apoptosis was measured by Cell Death ELISA. Results show mean values of each data point ± SE (n= 4).

We recently reported that 5-Lox inhibition-induced apoptosis in prostate cancer cells occurs via inhibition of PKCε without inhibition of Akt (32,33). Thus, we wanted to test whether WDL-induced apoptosis is also independent of Akt inhibition. We observed that treatment with WDL downregulates PKCε in a dose-dependent manner, but does not decrease phosphorylation of Akt in the same experimental conditions (Fig. 6A and B) which suggests that WDL induces apoptosis in prostate cancer cells via downregulation of PKCε but not via inhibition of Akt.

Though it is known that inhibition of 5-Lox induces apoptosis in prostate cancer cells (20–25), and that WDL inhibits the activity of 5-Lox (17), WDL is not a specific inhibitor of 5-Lox because at higher doses it inhibits IKKα, topoisomerase IIα, trypsin and PLA2 (8–13,34). Thus, we wanted to verify whether the apoptosis-inducing effect of WDL in prostate cancer cells occurs via inhibition of 5-Lox activity. Results are depicted in Fig. 6C showing that WDL-induced apoptosis in prostate cancer cells is effectively prevented by 5-oxoETE, a metabolic product of 5-Lox, whereas 15-oxoETE, a product of 15-lipoxygenase, was without effect. These findings suggest that the apoptosis-inducing effect of WDL in prostate cancer cells is mediated (at least partially) via inhibition of 5-Lox activity.

## Discussion

We observed that the natural compound WDL reduces viability of both androgen-sensitive (LNCaP) as well as androgen-independent (PC3, DU145) human prostate cancer cells, whereas it exerts only marginal effect on normal, non-cancer prostate epithelial cells (PrEC) in the same culture conditions (Fig. 1). These observations document for the first time that WDL possesses significant cancer-selective action, and suggest that WDL may be effective as a small molecule agent against prostate cancer. Our observation is of particular significance because it shows that WDL affects the viability of both androgen receptor-positive LNCaP (35), and androgen receptor-negative PC3 and DU145 (36,37) human prostate cancer cells with similar potency (IC_50_s of ∼8–12 *μ*M), suggesting that this effect of WDL is independent of androgen receptor status of these cancer cells. Herbal formulations of the source plants *Eclipta alba* and *Eclipta prostrata* have been used in India for centuries against liver damage caused by various hepatotoxins, for hair re-growth, for bronchitis and asthma, and for general well being as a rejuvenator (1–5). Crude extracts of plants contain numerous compounds, and the composition varies from sample to sample and on growth conditions of plants. However, WDL and demethylwedelolactone were identified as major components after fractionation of crude plant extracts, and are now available in pure forms for testing and mechanistic understanding (1–5,12,18). Both PC3 and DU145 cells were isolated from distant metastatic sites (bone and brain, respectively) and are androgen-independent (35–38). Thus, our observations open up the possibility of using WDL against deadly diseases such as androgen-independent metastatic prostate cancer for which currently there is no cure available.

A major advancement in our understanding about WDL as a pure compound is that it severely alters morphology and induces apoptosis in prostate cancer cells (Figs. 2 and 3). This apoptosis is associated with externalization of phosphatidylserine, cleavage of PARP, phosphorylation of H2A.X, and degradation of chromatin DNA to nucleosomal fragments. Cells undergoing apoptosis externalize phosphatidylserine which is characterized as a signal from dying cells for macrophage engulfment and clearance from the system (39). PARP is a protein substrate of executioner caspases and its characteristic cleavage is considered as an indicator of caspase-mediated apoptotic cell death (40). Degradation of chromatin DNA to nucleosomal fragments is considered as a hallmark of advanced stage of programmed cell death (41,42). Induction of apoptosis in cancer cells has been recognized as an effective approach to limit cancer growth because cancer cells are often observed to be endowed with increased capacity to prevent apoptosis, and pose resistance to chemo- and radiation-therapy (43–45). This is particularly important for prostate cancer because clinically prostate cancer is often characterized as slow-growing where anti-mitogenic therapies are not much effective. Thus, our observation of the induction of apoptosis not only adds a new dimension to the pharmacological properties of WDL but also opens up a possibility of using this agent to sensitize prostate cancer cells to undergo apoptosis.

Activation of the stress-activated protein kinase SAPK/JNK is a common, well-characterized cellular process for induction of apoptosis in various types of cells, and it was previously reported that 5-Lox inhibition induces apoptosis in prostate cancer cells via rapid activation of c-Jun N-terminal kinase (26–31). Thus, we examined whether WDL induces apoptosis in prostate cancer cells via activation of JNK. When prostate cancer cells were treated with WDL a rapid and strong activation of JNK occurred which was inhibited when cells were treated with inhibitors of JNK which also blocked induction of apoptosis, suggesting that WDL-induced apoptosis in prostate cancer cells is dependent on JNK activity (Fig. 4A and B). JNK modulates the function of mitochondrial apoptosis-regulating proteins and in turn induces permeability transition to release apoptosis-inducing factors (46,47). Our observation of WDL-induced damage of mitochondria which resulted in permeability transition and loss of membrane potential-sensitive dye suggests that WDL-induced apoptosis in prostate cancer cells involves JNK activation as well as loss of mitochondrial function (Fig. 4C).

Caspases are activated by both the mitochondrial and cell death receptor-mediated apoptosis pathways and play a causal role in the apoptosis process (48). Caspase-3 is one of the executioner caspases that is activated by upstream caspases, caspase-8 and -9. Numerous intracellular peptide substrates of the executioner caspases have been characterized including PARP, gelsolin, cytokeratin and endonuclease (49–51). As a first time report of apoptosis induction by WDL, we wanted to know whether activation of caspase-3 occurs in this type of apoptosis process, and whether caspase-3 activation plays any role in WDL-induced apoptosis in prostate cancer cells. Our analysis revealed that WDL treatment increases the enzymatic activity of caspase-3 in a dose-dependent manner (Fig. 5A). Moreover, it was observed that inhibition of caspase-3 by specific chemical inhibitor significantly prevents induction of apoptosis, suggesting that WDL-induced apoptosis in prostate cancer cells is caspase-dependent (Fig. 5B). This finding is similar to our previous observations of caspase-dependent apoptosis in prostate cancer cells induced by other 5-Lox inhibitors (26).

How WDL can induce apoptosis in prostate cancer cells is an intriguing question. A notable feature of WDL as a pure compound is that it is a potent inhibitor of 5-Lox (IC_50_=2.5 *μ*M) which inhibits 5-Lox activity by an oxygen radical scavenger mechanism (17,18). However, WDL is not a specific inhibitor of 5-Lox because it also inhibits other molecules at various concentrations (8–13,34). Previous studies have demonstrated an essential role of 5-Lox in the regulation of survival of both androgen-sensitive as well as androgen-independent prostate cancer cells (19–25), because inhibition of 5-Lox induces apoptosis in prostate cancer cells which is prevented by exogenous metabolites of 5-Lox (20,26,32,33). Thus, 5-Lox has emerged as a potential molecular target for therapeutic development against prostate cancer. However, potency, solubility, and cancer selectivity of several available 5-Lox inhibitors have limited their use for prostate cancer therapy. Based on published reports on the 5-Lox inhibitory effect of WDL, we expected that WDL, like other 5-Lox inhibitors, will decrease viability and induce apoptosis in prostate cancer cells via inhibition of PKCε (33) but without inhibition of Akt (32). Indeed we observed that WDL induced-apoptosis in prostate cancer cells is associated with dramatic inhibition of PKCε, whereas no inhibition of Akt was observed (Fig. 6A and B). Our observations of the induction of apoptosis in prostate cancer cells by WDL, and the prevention of apoptosis by 5-oxoETE (a metabolite of 5-Lox), but not by 15-oxoETE (a metabolite of 15-Lox) are consistent with the idea that the apoptosis-inducing effect of WDL in prostate cancer cells is mediated, at least partially, via inhibition of 5-Lox activity (Fig. 6C). Altogether, these findings indicate that WDL, a plant-derived coumestan compound, possesses significant anticancer properties, and suggest that it is possible to find newer 5-Lox-targeting agents from natural sources for development of effective therapy against prostate cancer.

Prostate cancer is the most common form of malignancy and second leading cause of cancer-related deaths in men in the United States (52). Though prostate cancer initially responds to anti-androgenic therapy, androgen-refractory disease almost always develops (53,54). Development of hormone-independent metastatic prostate cancer always ends up with a fatal outcome because currently there is no treatment available for this type of prostate cancer (54). Thus, novel agents and strategies are urgently needed to improve treatment options for androgen-independent prostate cancer. Based on the potency, solubility, and selectivity profile of WDL against metastatic prostate cancer cells *in vitro*, it appears that WDL is a novel, promising candidate drug and should be tested further for the treatment of both androgen-sensitive as well as androgen-independent prostate cancers.

## Abbreviations:

WDL: wedelolactone;
5-Lox: 5-lipoxygenase;
PKCε: protein kinase C ε;
5-oxoETE: 5-oxoeicosatetraenoid;
PARP: poly-ADP ribose polymerase;
IAP: inhibitors of apoptosis;
ELISA: enzyme-linked immunosorbent assay;
FITC: fluorescein isothiocyanate
